# Physiotherapy Intervention Focusing on Proprioceptive Retraining for Tibial Pseudoarthrosis Patients Operated With Ilizarov External Fixator: A Case Report

**DOI:** 10.7759/cureus.52274

**Published:** 2024-01-14

**Authors:** Anushka Rohankar, Vaishnavi M Thakre, Maithili M Deshpande

**Affiliations:** 1 Musculoskeletal Physiotherapy, Ravi Nair Physiotherapy College, Datta Meghe Institute of Higher Education and Research, Wardha, IND

**Keywords:** tibia, pseudoarthrosis, rehabilitation, physiotherapy, ilizarov’s fixator, case report

## Abstract

Pseudoarthrosis of the tibia is an uncommon condition that occurs due to non-union of bone and typically requires surgery. It may cause fractures that develop spontaneously or after minor trauma. Physiotherapy is an excellent treatment for this uncommon condition. The tibia of the affected limb becomes malformed and bends backwards. This case report outlines the interdisciplinary programme adopted to successfully manage a 23-year-old female patient with right-sided tibial pseudoarthrosis. Following the implantation of Ilizarov's external fixator to correct the deformity, the patient was referred to the physiotherapy department for post-operative management. After the implementation of a tailor-made physiotherapy protocol focusing on proprioceptive retraining, significant improvements were seen in her joint proprioception, muscular strength and range of motion.

## Introduction

When two bones do not properly fuse, it is known as pseudoarthrosis [1]. The Crawford classification is one of the most widely used, in which type 1 includes anterior bowing, more dense cortical tissue, as well as a thin medulla; type 2 includes anterior bending, sclerotic bone, and a tiny medulla; type 3 includes anterior bending with cyst or pre-fracture symptoms; and type 4 includes anterior bowing, clearly visible fracture, and tibial and fibular pseudoarthrosis. The pseudo-oligotrophic arthrosis, artificial arthrosis, or hypotrophic pseudoarthrosis (non-vital) are the various types of pseudoarthrosis [2]. Congenital pseudarthrosis of the tibia (CPT) is a rare condition that can manifest in an array of clinical presentations, from total non-union with severe bone abnormalities to simple anterolateral tibial angulation [3]. The pathogenesis of this condition demonstrates either lower osteogenic capability of the bone due to deficiencies in the local ability to vascularize the bone or greater osteoclastic action due to abnormalities in signalling [4].

Various surgical approaches can be used to treat CPT, such as the cross-union approach, intramedullary rods or nails, vascularized fibular grafting, Ilizarov ring fixation, or a combination of two or three treatments. Regardless of the approach selected, surgery involves use of the same biological principles to rectify angular deformity, bone bridging of the defect, and pseudarthrosis excision [5]. However, residual difficulties are prevalent. Furthermore, residual challenges discovered following primary union may occasionally be severe enough to compromise the functioning of the affected limb. To achieve a functional, stable extremity at the end of treatment, physiotherapy rehabilitation should be given [6,7]. Immediate complications include direct injury to neurovascular structures. Early complications include bleeding, pain that can result in a compartment syndrome or haematoma, pulmonary embolism, deep vein thrombosis, and nerve injury as a result of straining [8].

Careful physiotherapy is required to avoid subluxations and joint contractures, which occur in this situation as a consequence of muscle irritation resulting from cables or pins piercing them. Stretching and maintaining range of motion (ROM) are critical for preventing contractures, dislocations, and subluxations, while functional loading and ambulation are required for ossification of the regenerating bone, emphasizing the importance of physical therapy in the success of Ilizarov's procedure [9]. Due to prolonged immobilization within the Ilizarov ring fixator, proprioceptive loss is also a major complication. Currently, limited available literature regarding physiotherapy for patients having Ilizarov is available and no potential studies in the area of Ilizarov rehabilitation with respect to proprioceptive loss have been carried out. Therefore, our aim was to evaluate the effectiveness of comprehensive physiotherapy management incorporating proprioceptive neuromuscular facilitation to improve proprioception and reduce other postoperative complications.

## Case presentation

**Figure 1:**
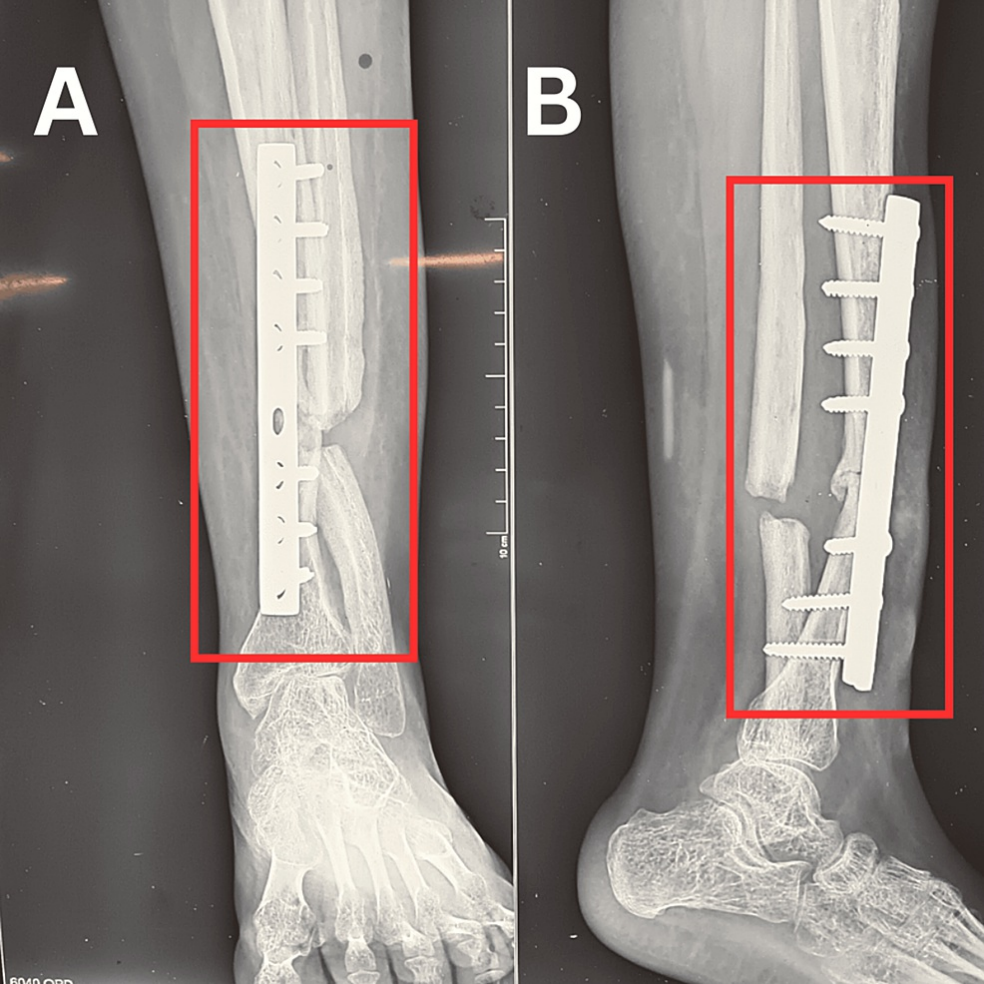
X-ray of right tibia with implants Rectangle represents open reduction internal fixation done by nailing and plating

**Figure 2:**
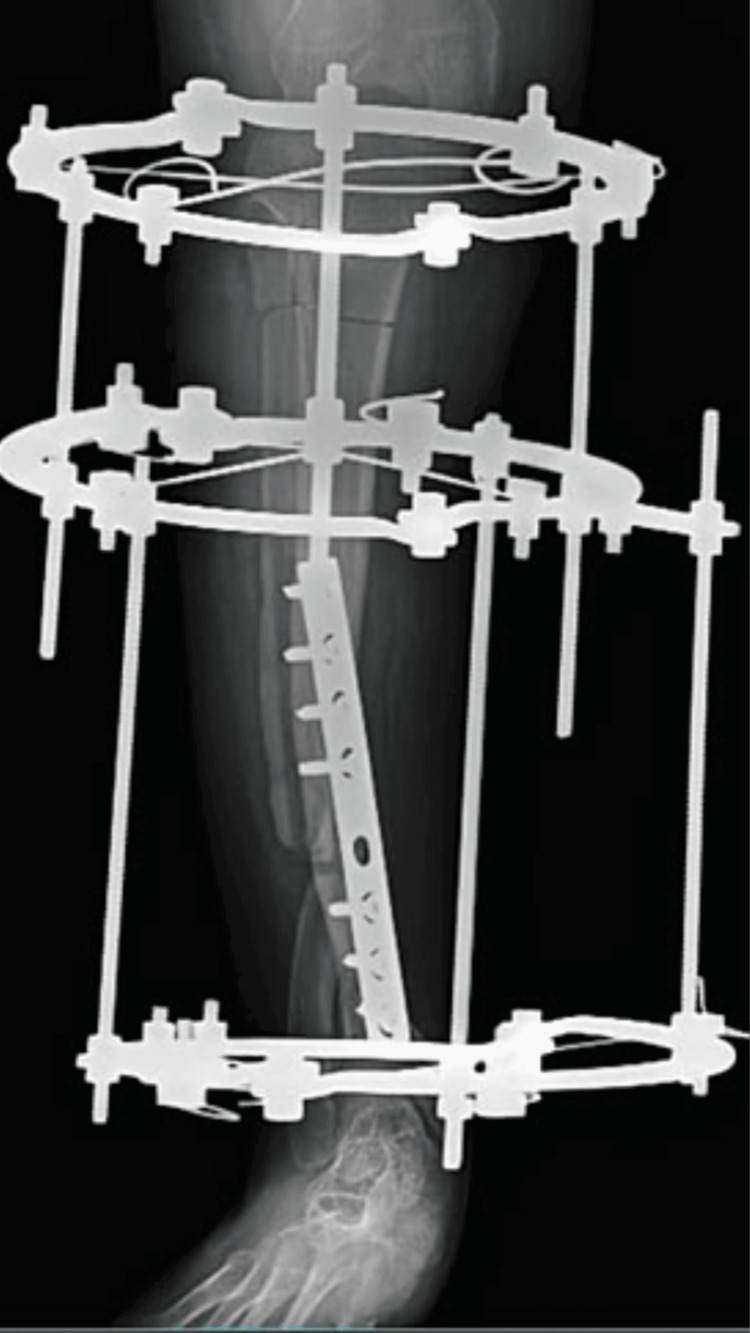
X-ray of right tibia showing Ilizarov external fixator

We have presented a case of a 23-year-old female who came to our tertiary care hospital with complaints of pain and swelling over her right lower limb since one month. She is a known case of congenital tibial psuedarthrosis with valgus deformity since 20 years. She was operated for correction of deformity one year back with open reduction internal fixation and plate osteosynthesis but the surgery was unsuccessful and valgus deformity persisted. Patient was apparently alright one month back when after which she started having pain over right leg. Pain was insidious in onset and non-progressive. It was moderate in intensity and constant through out the day. It aggravated with movement, walking, climbing stairs and relieved with rest. Patient also complained of swelling over right leg which was sudden in onset, progressive in nature. Swelling aggravated with movement, walking, climbing stairs and relieved with rest. For the above complaints patient visited our tertiary care hospital where she was managed with an Ilizarov external ring fixator and was referred to physiotherapy for further management. Figures 1, 2 show X-rays of the patient before and after Ilizarov external ring fixation respectively.

Clinical findings

Prior to commencing the examination, the patient's informed consent was taken. The patient was conscious, cooperative, and able to follow instructions and had a mesomorphic build. The patient underwent assessment while lying supine. Upon examination of the right leg, generalized swelling observed over the upper portion of the leg and knee. The ilizarov external ring fixator was present extending from knee joint till ankle joint. Upon palpation, the local temperature was within normal limits. Tenderness was detected over the upper portion of the left leg, specifically over the tibial condyles. There was reduced muscle strength and painful restriction in the ROM in the right knee and ankle joint. Patient rated pain as 4/10 on rest according to the numerical pain rating scale.

Therapeutic intervention 

As needed for her recovery, the therapeutic intervention had been discussed with her parents. The goals of the treatment were decided. The physiotherapist determined the progression of the intervention, which was carried out five times a day (Table 1).

**Table 1:** Physiotherapy intervention Dosage for PNF [10]. PNF: proprioceptive neuromuscular facilitation, NA: not applicable, reps: repetition

Goals	Therapeutic intervention	Dosage
To prevent muscle tightness	Positioning	NA
Pain reduction	Cryotherapy	In circulation motion 5 to 10 minutes
To improve the stability of the knee and reduce the risk of injury	PNF rhythmic stabilization technique	10 reps of 3 sets with 10 seconds hold
To improve lower limb muscle strength	Isometrics strengthening exercises for bilateral lower limb	10 reps of 3 sets with 5 seconds hold
To improve joint proprioception	PNF contract-relax and hold-relax technique	10 reps of 3 sets with 10 seconds hold
To increase the range of motion of lower limb	Active range of motion exercises for lower limb within pain-free range	10 reps of 3 sets
To regain the normal gait pattern and initiate ambulation.	Ambulation with walker	NA

Follow-up and outcome measures

The outcome measures were assessed after four weeks of physiotherapy and later on after removal of the Ilizarov external ring fixator which was done four months after operation and the patient showed improvement. Tables 2-4 show the pre- and post-treatment scores of outcome measures.

**Table 2:** Manual muscle test of lower limb MMT: manual muscle testing 0: No contraction; 1: Flickering of contraction; 2: Full range of motion in gravity-eliminated position; 3: Full range of motion against gravity position; 4: Full range of motion against minimal resistance; 5: Full range of motion against maximal resistance

Muscle group	Pre-treatment MMT grade	Post-treatment MMT grade (After removal of ilizarov external fixator)
Plantar flexors	2/5	4/5
Dorsiflexors	1/5	3/5
Investors	1/5	3/5
Evertors	1/5	3/5
Knee flexors	3/5	4/5
Knee extensors	3/5	4/5
Hip flexors	3/5	4/5
Hip extensors	3/5	4/5
Hip internal rotators	3/5	4/5
Hip external rotators	3/5	4/5
Hip abductors	3/5	4/5
Hip adductors	3/5	5/5

**Table 3:** Range of motion right lower limb ROM: Range of Motion, NA: not assessable

Joint	Movement	Pre-treatment ROM	Post-treatment ROM (after removal of Ilizarov external fixator)
Ankle	Plantarflexion	0^o^	20^o^
	Dorsiflexion	0^o^	15^o^
Knee	Flexion	NA	90^o^
	Extension	NA	15^o^
Hip	Flexion	40^o^	90^o^
	Extension	15^o^	20^o^
	Abduction	30^o^	40^o^
	Adduction	10^o^	15^o^

**Table 4:** Pre and post-physiotherapeutic rehabilitation outcomes measures pre-treatment ROM ROM: Range of Motion

Scale	Pre-treatment	Post-treatment (after removal of Ilizarov external fixator )
Lower Extremity Functional Scale	20/80	60/80
Functional Independence Measure	30/126	100/126

## Discussion

For CPT, no standardized surgical technique has been identified. For these patients, preventing refracture, achieving union and correcting deformities-especially those affecting the ankle joint are the main goals of treatment. Clinical practice employs a variety of therapeutic modalities, such as Ilizarov with intramedullary fixation, Ilizarov alone, and free vascularized fibular graft [11,12]. In this case report, we saw the comprehensive physiotherapy rehabilitation involving proprioceptive neuromuscular facilitation (PNF) in the patient with congenital tibial pseudoarthrosis operated with an Ilizarov external ring fixator. In this study we have given isometric strengthening exercises, range of motion exercises, proprioceptive exercises and gait training and there was significant improvement in lower limb muscle strength, range of motion, weight-bearing and proprioception of joints. The case study carried out by Mundada et al. describes an extensive physiotherapy rehabilitation program for patients undergoing an Ilizarov ring fixator and sequestrectomy for chronic osteomyelitis. In this instance, there was a significant improvement over the course of the eight weeks of rehabilitation, including a safe return to normal activities, improvements in ROM, muscular strength, pain relief and gait, and prevention of secondary impairments [13].

Our focus was also on regaining joint proprioception which was lost due to the prolonged immobilization in Ilizarov external fixator, hence we have incorporated PNF techniques such as rhythmic stabilization, hold-relax and contract-relax and found the improvement in joint proprioception leading to improved lower extremity function which was evaluated on lower extremity functional scale. Ferreira et al. carried out a similar study involving proprioceptive retraining in patients with bicondylar tibial plateau fractures treated with the Ilizarov circular external fixator and found good functional outcomes [14]. 

The best approach to surgery based on fundamental treatment principles is necessary to minimise post-primary healing challenges. Regular passive motion exercises over an extended period of time is a useful technique to enhance postoperative therapy and greatly improve the range of motion at both periods of follow-up [15]. Since the Ilizarov approach has produced good outcomes, it may be regarded as an advantageous and effective therapy for recurring or ignored deviations [16]. Physiotherapists with varied degrees of experience treating individuals using an Ilizarov fixator provided their comments for the study [17]. Although it is possible to argue that these individuals need standard, high-quality musculoskeletal rehabilitation, the possibility of associated soft tissue difficulties results in a unique population that needs additional consideration and research [18].

## Conclusions

Tibial congenital pseudoarthrosis is an uncommon disorder. In order to prevent secondary complications, it can be managed surgically and with appropriately tailored rehabilitation. In this case report we saw the patient having pseudarthrosis of the tibia treated with an Ilizarov fixator. There was a substantial increase in function over the course of four weeks of rehabilitation, including pain relief, range of motion, improvements in muscular strength, joint proprioception and gait, as well as prevention of subsequent impairments that eventually facilitated a safe return to routine activities. 
